# Teachers’ Constructivist and Ethical Beliefs

**DOI:** 10.3390/bs10060096

**Published:** 2020-05-29

**Authors:** Marie Sharkey, Hugh Gash

**Affiliations:** 1 English Department, British International School, Ajman PO Box 8811, UAE; mary@bisuae.ae; 2 Institute of Education, Dublin City University, D09 Y18 Dublin, Ireland

**Keywords:** teachers, beliefs, constructivism, ethics, idealism, relativism

## Abstract

Teachers’ approaches and beliefs are key determinants of teachers’ practice. This study was designed to examine whether two aspects of Irish primary teacher beliefs are associated, their views on constructivist practices and their views on two ethical dimensions (idealism and relativism). The views of a sample of 35 teachers were assessed using the Constructivist Learning Environment Survey (CLES) and the Ethical Position Questionnaire (EPQ). Significant relationships were found between ethical position and scores on dimensions on the CLES. For example, idealistic teachers valued uncertainty and student negotiation more than teachers with high relativist scores. The results are discussed in the context of continuing professional development and future research.

## 1. Introduction

In the Irish Republic, Piaget’s child centered approach has been central to the primary school curriculum since 1971 [1]. In psychology, the successor to Piaget’s theory is constructivism. Changing from traditional didactic teacher driven teaching to methods that emphasize the child’s learning is a process that involves letting go of some goals and adopting others. There are important educational issues concerning the engagement of children in the educational process on the one hand and ensuring that educational standards and goals are guaranteed on the other. Some school systems prioritize measurable gains in achievement and others emphasize children’s engagement and the processes, both social and individual, that are essential to this [2]. Constructivism is central in this debate and these issues were well presented in a book edited by Sigmund Tobias and John Duffy “Constructivist Instruction: Success or Failure?” [3]. The interplay of ideas and beliefs in this debate is a type of ecology of teaching and learning [4].

Constructivist theory has been applied in educational settings with varying emphases over the past 40 years or so [5]. Central to this development is the idea that intelligence organizes the world by organizing itself. What has always been difficult about this theory is its prioritization of interpretation. Meaning making is private and personal, at least initially. From jokes to gravitational waves, people can speak coherently and intelligently but listeners may or may not understand. Education plays an important role in communicating meaning and so contributes directly to children’s social and personal development. Educational processes in turn are a matter for Governments, for schools, for teachers and for children. Indeed, the variety of influences on aspects of teaching varies from context to context so that their systemic nature is inevitable. This paper is about how two dimensions of teacher thinking influence the educational process: teacher belief and constructivism.

The constructivist approach to epistemology that became well known as Piaget’s theory moved into mainstream psychology in the 1960s. In the United States, John Flavell [6] wrote an introduction to Piaget, and this holistic approach became a serious rival to the behavioral approach that had been dominant in the 1950s. In linguistics, a similar move to the holistic approach emerged with Noam Chomsky [7]. Two different types of educational context have facilitated research into educational applications of constructivist theory: social inequality and the digital revolution.

In the 1960s educators in the United States were encouraged to look for suitable theoretical models to combat educational disadvantage. Head Start was the initial big educational and social intervention program in the USA in the 1960s [8,9]. The limited short-term educational success of the Head Start program led to a Follow Through program, in which various educational approaches were used and evaluated very thoroughly. The approaches included basic skills models, cognitive/conceptual and affective/cognitive models. One cognitive program was the Mathemagenic Activities Program at the University of Georgia, directed by Charlie Smock, where Ernst von Glasersfeld launched radical constructivism [10]. It is beyond the scope of this paper to analyse the Follow Through programs in detail, but some findings support our aim to study teacher beliefs. One initial finding was that the skills approaches taught skills well, and better than the child centered approaches. However, critics pointed out that the various approaches were not easy to categorize and that the effectiveness of any approach varied from one school district to another [11]. In other words, teachers vary enormously in their effectiveness whatever approaches and measures are used. There are more specific and recent evaluations on the effects of constructivist programs [3]. It is clear that how teachers teach and so how they think about teaching is critical to how they work. This was highlighted again in a recent study on Growth Mindset [12,13]. Turning to the digital revolution, recently Governments in many countries have been concerned to promote science and technology in schools [14,15]. This work has provided an opportunity to see how teachers thinking about constructivist approaches varies in different countries [16]. Countries also vary of course in the extent that commercial and educational interests mingle. However, in Ireland this marriage of the commercial and educational was celebrated with the provision of internet access by Bord Telecom as part of the Irish Department of Education’s IT2000 initiative [14]. In this context teacher thinking in constructivist approaches that used digital learning were investigated in European Funded projects under the Comenius Programme [17].

The following section considers in more detail central features of the constructivist approach as they apply to education and how they can be measured. One central feature of constructivism is the idea that what we know is a personal construction and that we may differ in how we make these constructions. This is why we differ in our beliefs as to what is important in education, the process or the product for example. It may also be the case in our ethical beliefs.

Constructivism emphasizes that people make their understandings of the world and that these may be different [10]. This model has educational implications [3] and also the ethical implication [18] that people are responsible for the way they interpret their experience. The aim of this study is to look for a relation between a sample of teachers’ views about dimensions of constructivist teaching and their ethical orientation. A second objective is to understand variability in teacher belief within the constructivist framework.

## 2. Constructivism and Education

Jean Piaget [19,20] described the process of thinking in a way open to the radical constructivist interpretation proposed by Ernst von Glasersfeld [10]. Piaget’s process of equilibration included assimilation and accommodation (the internal adjustment of “rules” to experience in meaning making). Assimilation has sometimes been interpreted to mean “taking in information” from the environment [21]. Glasersfeld [10] emphasised that all cognitive processing is internal, so the child is always interpreting and tries to make sense on the basis of what she knows. In this context, assimilation is a filtering of “reality” by what is experienced and direct experience of “reality” is an illusion. We experience (sense data) and interpret. However, as was clear in John Dewey’s [22] approach, our minds use experimental processes to optimize our understanding of experience. The constructivist teacher’s role is to facilitate this process and to respect it in all educational activities.

Teachers vary as to whether they think giving information is very important, or whether child learning is paramount [23]. Therefore, this radical aspect of constructivism does not sit well as a model of teaching to many teachers. The issue of the meaning of “reality” and the role of truth are two centrally related issues in constructivism [10]. Education is an important social activity. Within the educational community, there have been a variety of approaches to constructivism. Social constructivism prioritizes the social processes that are paramount for teachers. Radical constructivism reminds us that we cannot extend our knowledge beyond our experience with certainty [24]. We do our best, but we can always be surprised by a gap between what we think we know and what we experience. The existence of this gap is a source of creativity, if it was absent, learning would only be about memorizing.

Constructivism requires that we are sensitive to the different interpretations people put on events. Our interpretation may be different from a child’s or from any other person’s interpretation, and this applies to what we are teaching and to the social context we are trying to create. If we are teaching we try to understand what the learner has understood and not understood, and how best to help the learner be involved in learning [5]. Yes indeed, the social processes are vital, but it is the learner who either “sees” or “doesn’t see” what we are trying to teach. The learner decides what she learns and whether she likes what she is learning. This, of course means recognizing that what we know is as personal as our appearance, we have come to know and feel about certain things and not know and feel about other things, and we should be sensitive to the each other’s processing [18].

*Teacher’s beliefs about constructivism.* In an early study about student teachers’ implicit epistemological beliefs with a sample of 165 students at St Patrick’s College in Dublin, Gash [25] found males and females differed on a number of the questions asked. The Irish male students were much more likely to agree (71%) than females (15%) that “The history of science shows that knowledge acquisition occurs in jumps and there are shifts in the way problems are approached” and the same pattern occurred for the statement “Some knowledge is innate” (males 78%, females 23%). In the early 1990s, [26] developed a twenty-five item Constructivist Learning Environments Survey (CLES) to examine key dimensions of constructivist learning theory:

This instrument was in response to the need of researchers to “assess the degree to which a classroom environment is consistent with a constructivist epistemology, and to assist teachers to reflect on their epistemological assumptions and reshape their teaching practice” [26] (p. 3). The original 1991 version examined a “psychosocial view of learning that focused on students as co-constructors of their own knowledge” [27] (p. 2). For this, it assessed classroom environments for student negotiation, student prior knowledge, student autonomy and student centeredness. However, although this view was consistent with constructivist teaching, the authors thought it failed to acknowledge the cultural context framing the classroom environment.

Further research [23] revealed that invisible forces such as powerful cultural myths “rooted in the histories of science or mathematics, and of schooling” could counteract the development of constructivist learning environments. The CLES was therefore redesigned by [23] to incorporate a “critical theory perspective on the cultural framing” of the classroom learning environment. Their rationale for the change was based on the “importance of teachers and students becoming critically aware of how their teaching and learning roles are being unduly restrained by … otherwise invisible forces” [23] (p. 293). These forces included the positivist/empirical view of the importance given to transmission of knowledge by teachers, and the notion of scientific theory being static and not socially constructed. These authors also state that the original CLES version was based on a theory of constructivism influenced at the time by ‘conceptual change’ research which promoted teachers’ facilitation of conceptual development in students.

This conceptual-change research highlights the key role of students’ prior knowledge in their development of new conceptual understandings, and the reflective process of interpersonal negotiation of meaning within the classroom community. Thus, at that time, ‘student negotiation’, ‘prior knowledge’ ‘rectifying scientific and mathematical misconceptions’ and students as ‘co-constructors of their own knowledge’ became the focus of constructivist transformation in schools. The authors state that studies have shown that traditional-style, teacher-centered classrooms could “readily assimilate” these aspects of constructivism but still “remain largely unchanged” [23].

Cultural restraints, therefore, were not assessed by the original version, thus the redesigned CLES incorporated a “critical theory perspective on the cultural framing of the classroom learning environment” [23] (p. 294). Like other instruments used to measure different aspects of student school-life, such as ‘satisfaction’, ‘difficulty’ and ‘involvement’, the original version of the CLES did not enable a study of the ‘revolution’ in science and mathematics education [28]. Instead, it gave opportunities to assess and refine classroom environments which remained consistent with the traditional pedagogical paradigm, in which the central role of the teacher and student remained more or less the same: transmitter and recipient. Radical changes were made, therefore, in order to include the ‘critical’ dimension which had been missing: ‘the role of the teacher and student’-‘critical voice’ and ‘shared control’.

*Critical constructivism: The new CLES.* Each scale of the new version of the Constructivist Learning Environment Survey (CLES) [23] was designed to obtain measures of students’ perceptions of the frequency of occurrence of five key dimensions of a critical constructivist learning environment: personal relevance, uncertainty, critical voice, shared control, and student negotiation. The CLES contains 25 items altogether, with five items in each of the five scales. Response alternatives for each item are “Almost Always”, “Often”, “Sometimes”, “Seldom”, and “Almost Never”. Details on this questionnaire are provided in Appendix A.

The personal relevance scale focuses on the connectedness of school science to students’ out-of-school experiences, and with making use of students’ everyday experiences as a meaningful context for the development of students’ scientific and mathematical knowledge. The uncertainty scale assesses the extent to which opportunities are provided for students to experience scientific knowledge as arising from theory-dependent inquiry involving human experience and values, and as evolving, non-foundational, and culturally and socially determined. The critical voice scale examines the extent to which a social climate has been established in which students feel that it is legitimate and beneficial to question the teacher’s pedagogical plans and methods, and to express concerns about any impediments to their learning. The shared control scale is concerned with students being invited to share control of the learning environment with the teacher, including the articulation of learning goals, the design and management of learning activities, and the determination and application of assessment criteria. Finally, the student negotiation scale assesses the extent to which opportunities exist for students to explain and justify to other students their newly developing ideas, to listen attentively and reflect on the viability of other students’ ideas and, subsequently, to reflect self-critically on the viability of their own ideas.

1. Personal relevance (learning about the world)
2. Uncertainty of science / nature of science (NOS)
3. Critical voice (learning to speak out)
4. Shared control (learning to learn)
5. Student negotiation (learning to communicate)

## 3. Examining Teacher Ethical Beliefs

A second questionnaire measuring teacher belief/ideology was also used, namely, Forsyth’s [29] Ethics Position Questionnaire (EPQ) which is designed to measure ethical ideology along two dimensions, relativism and idealism. This questionnaire, consisting of 20 Likert-scale items, was also presented to the teachers with the intention of exploring their broader belief structures. Details on this questionnaire are provided in Appendix A. The EPQ assesses an individual’s “degree of idealism and rejection of universal moral rules in favour of relativism” (p. 175). Item and factor analysis were used throughout its development, and the two scales possess adequate psychometric properties, moderately high internal consistency, reliability, and discriminant validity. The EPQ “does not classify ethical ideologies solely on the basis of their being “principled” (p. 183) or moral maturity, unlike the Kohlberg model, and is therefore recommendable as a more general typology. Forsyth [29] adds that the idealism-relativism classification may be useful to focus on the moral judgements of adults.

The aim of the present study was to examine whether the ethical relativists would endorse the uncertainty in constructivism, or whether the ethical idealists would enthusiastically endorse the challenge of change involved in this approach to teaching and learning.

## 4. Method and Sampling

Convenience and snowball sampling were used due to time and cost restrictions (Cohen, Manion & Morrison, 2011). The sample consisted of 50 primary teachers working in rural schools who were given the two questionnaires in the context of Dublin City University’s ethical guidelines. All teachers gave their informed consent to inclusion before they participated in the study. The study was conducted in accordance with the Declaration of Helsinki, and the protocol was approved by the Ethics Committee of St Patrick’s College Dublin at the beginning of the study(letters to teachers dated 27 November 2013 [30]) Thirty five questionnaires were returned. There are five scales in the CLES questionnaire. Each scale has five questions with Likert scale answers going from almost always (5), often (4), sometimes (3), seldom (2), and almost never (1). Respondents’ answers were summed to give a score for each scale in Table 1 and Table 2 below. Similarly the Ethics Position Questionnaire (EPQ) consists of 20 items, 10 to measure ethical relativism and 10 to measure absolutism with Likert scale answers going from 1 = disagree strongly, 2 = disagree slightly, 3 = neither agree nor disagree, 4 = agree slightly, 5 = agree strongly. Copies of each questionnaire the CLES and the EPQ are included at the end of the paper.

The reliabilities of the scales and the correlations between all seven scales were checked. As described in the next section and following initial data analysis, participants were divided into two groups (high and low on each ethical dimension) to compare these groups on the five CLES constructivist scales. The high relativist and the high idealist groups were each composed of the participants with scores in the top 25%. SPSS 20 was used to calculate statistical values. Means of the five CLES variables were compared using t-tests with independent samples (high and low groups on the EPQ).

## 5. Results and Discussion

The CLES [23] and the Ethics Position Questionnaire (EPQ) [29] have each been shown to be reliable in previous studies. In the present study Cronbach’s alpha for the CLES and the EPQ Idealism scales was over 0.90 and for the EPQ Relativism scale 0.85.

The present study is about the relation between teachers’ beliefs in two domains, and so differed from a study by Gash and McCloughlin [31] in that it does not compare groups of teachers that differ in country or origin or in the grade level they teach. Rather, the question here is whether and how the types of belief examined (two ethical and five constructivist) are related.

As a first step a correlation analysis of the two sets of scales showed that the five constructivist scales were not significantly related to ethical relativism. However, the ethically idealist scale correlated significantly and positively with two constructivist scales, uncertainty/NOS (r = 0.59, *p* < 0.001) and student negotiation (r = 0.41, *p* < 0.05). In addition, the idealism scale and the relativism scale correlated negatively (−0.48, *p* < 0.01).

Positive correlations between ethical idealism with these two dimensions of constructivism, especially student negotiation, might be expected in primary classrooms as it appears to be the easiest of the five dimensions to implement [31]. Gash & McCloughlin [31], using the same CLES in a 5-nation European study, found that Irish primary-school teachers had scores significantly higher on student negotiation than secondary teachers (*p* < 0.001). Furthermore, their findings showed that Ireland’s primary teachers had scores significantly higher than secondary teachers (*p* < 0.001) on personal relevance, which Marie Sharkey [30] found was not significantly correlated with idealism. Nevertheless, if two dimensions are related to ethical idealism it provides teachers with a way to begin self-reflection and group discussion, to develop the other three dimensions using the EPQ and CLES questionnaires in professional development contexts.

Given these correlations we were concerned to see if groups of teachers with different ethical approaches were different on the five dimensions of constructivism. To achieve this, we divided the teachers into groups based on their scores on ethical idealism and relativism. The range of scores on the idealism scale was from 18 to 49, and 25% of these had scores of 44 and over. Two groups of idealist teachers were formed (high and low), with those having scores of 44 and over being in the high group. In the case of the relativism scale the range was from 14 to 46, and 25% had scores of 39 or more. Two groups of relativist teachers were created (high and low) again using the top 25% of the sample as the high scoring group. Contrasting high scoring and low scoring groups for ethical idealism and ethical relativism, the teachers were compared on their sets of scores on the CLES.

## 6. Idealism and Aspects of Constructivism

Table 1 shows that the 10 teachers in the High Idealism category have mean scores of 2 to 4 points higher for each of the five constructivist scales than the 25 teachers with low scores on idealism. These differences are all significant (*p* < 0.05). Secondly, their mean scores on the two dimensions of constructivism, uncertainty (NOS) and student negotiation (SN), which correlate significantly with idealism in the first analysis above, indicate that high idealists have mean scores a full 4 and 3.3 points, respectively, above low idealists on each of the two dimensions. The 10 teachers with high Idealism scores, therefore, might be encouraged to engage in action research in their schools [31] in order to develop over time the three aspects of constructivism, PR, CV, and SC, which do not correlate significantly with idealism at the moment [32].

## 7. Relativism and Aspects of Constructivism

Conversely, Table 2 compares groups of relativist teachers (high and low) on constructivism shows that those low on relativism are more favorably disposed towards constructivist practice than their high relativistic counterparts. The difference in mean scores between high and low relativists ranges from 2 to 6 points and are all significant (*p* < 0.05). Uncertainty/nature of science, a central aspect of constructivism, having the lowest mean score at 7.67 amongst high relativists.

Secondly, in Table 2 we note that ‘shared control’, that is, learning involving student participation in lesson planning and assessment, shows a low mean of 13.67 for high relativists. A move to frequently using this dimension, which is a strong predictor of student interest in science [32], might also prove to be a difficult shift for a high relativist teacher in terms of change in student–teacher relations and teacher autonomy. High relativists, therefore, do not like constructivist claims that the study of science (NOS) is subjective rather than objective, that it involves uncertainty, and that scientific theory is socially constructed through “modelling, arguing and evaluating” [31] (p. 5) rather than being foundational and predetermined. This view may be contrary to that person’s central belief structure [33]. This central belief or ethical stance may, therefore, prove difficult to change, but may not be impossible with the teacher’s awareness being heightened through this sort of research.

Furthermore, if low-relativist figures in Table 2 are compared with low-idealists results in Table 1, we see that low relativists are slightly higher than low idealists on all five dimensions of constructivist practice. This finding illustrates that there may be combinations of relativism and idealism worth pursuing further, both statistically and in professional development.

Recalling that ethical idealism and ethical relativism correlate negatively, these results are not surprising and show the relevance of these ethical beliefs for constructivist practice. The main impact of these findings is that they show how these ethical beliefs are associated with teachers’ beliefs about constructivism. People who are high on idealism and low on relativism are more likely to think about constructivist practice favorably. In addition, the data show variability in the teachers’ acceptance of constructivist practice. While we might have predicted this from observation and discussion with teachers, these questionnaires provide tools on which to base discussion about classroom practice, so providing teachers with a means to reflect on what they do and what is best for learning in their classrooms.

These results may be usefully considered as feeding into the dimensions of teacher development described by Conway and Clarke [34]. They based their study on Fuller’s model of teacher development that shows a shift in concern from self, to tasks, to concerns about student learning. In an interview study, they report that beginning student teachers initially moved in an outward journey concerning self to tasks to students, but they then moved in an inward journey to consider how to grow as a teacher and a person. Discussions about how teachers think about the process of learning and how we relate to our students are central to this development.

These data show a relationship between beliefs and practice. That is, in this sample, these teachers’ beliefs influence their practice. This is shown by the statistically significant correlations existing between Idealistic beliefs and 2 aspects of constructivist practice (student negotiation and nature of science), and also by the significant influence of ethical idealism and ethical relativism on the five aspects of constructivist practice in the CLES.

Issues prompting this research include those highlighted in the NCCA [35] report on science education in Ireland. This report found that the enquiry-style curriculum, a product of reform imperatives to promote science in schools, is not being implemented as planned. Murphy et al. [36], and Varley et al. [37] found similar results, as did international studies cited in NCCA [35] and Gash and McCloughlin [31]. Text-book usage [38], lack of teacher content and pedagogical knowledge [39], and lack of teacher confidence [35] were seen as stumbling blocks to implementation of enquiry approaches. Teachers’ beliefs [40,41] are also viewed as major impediments to the change of teaching approach necessary for constructivist-curriculum implementation. The development of teachers’ awareness of the effects of their beliefs on instruction and student outcomes is crucial, [33,42]. Beliefs are considered to be the highest predictors of the actions people take in their lives [43], while in this sample, idealistic beliefs are the highest predictors of constructivist practice.

A variety of studies have used the CLES with teachers [31,41,43,44]. One feature they all have in common is that they show that teachers vary considerably in their acceptance of constructivism. Our experience of introducing teachers and student teachers to constructivism is that it takes time to construct a deep understanding of constructivism. This study shows how constructivism is connected with ethical beliefs. It is part of the meaning of constructivism that it has ethical implications [18], and it is in the nature of constructivism that one’s understanding of it is itself a process of construction [5]. In this paper, we have suggested that these questionnaires, the CLES and EPQ, might help teachers in their professional development. We acknowledge the small sample size as limiting the generalization of the results; however, we suggest that the relationships found are suggestive.

## 8. Conclusions

We have demonstrated a significant correlation between ethical idealism and two aspects of constructivist beliefs (uncertainty/knowledge of science and student negotiation/learning to communicate), In addition, further analysis of these data showed that high scores on ethical idealism (and low scores on ethical relativism) were associated with high scores on each of the five constructivist scales on the CLES. These are personal relevance (learning about the world); uncertainty of science/nature of science (NOS); critical voice (learning to speak out); shared control (learning to learn); and student negotiation (learning to communicate).

While the dimensions of teacher belief in constructivist practice are well established variables that differentiate teachers reliably, this study, although the numbers are small, established a relation between these constructivist variables and an ethical aspect of teacher belief. That is, a tendency to idealism in ethics extends to considering these constructivist variables as ideal. We note that the Irish Curriculum [35] is based on constructivism so these results may be culturally biased. Perhaps the best way forward is to engage in a community of practice such as described in Corbett [45], with a view to preparing for a larger scale empirical study. Our hope is that these dimensions would help establish a metric to help understand variation in teacher program implementation in appropriate intervention studies.

## Figures and Tables

**Table 1:** High (2) and low (1) idealism, and aspects of constructivism.

		1.	2.	3.	4.	5.
		Personal Relevance (PR)	Uncertainty of Science (NOS)	Critical Voice (CV)	Shared Control (SC)	Student Negotiation (SN)
1.00	Mean	19.32	11.60	17.84	14.92	19.08
	N	25	25	25	25	25
	Std.Deviation.	2.868	3.753	3.555	3.651	2.448
2.00	Mean	21.10	15.60	20.20	17.00	22.40
	N	10	10	10	10	10
	Std.Deviation.	2.644	4.033	3.521	3.944	2.503
Total	Mean	19.83	12.74	18.51	15.51	20.03
	N	35	35	35	35	35
	Std.Deviation.	2.885	4.196	3.657	3.799	2.864

**Table 2:** High (2) and low (1) relativism and aspects of constructivism.

		1	2	3	4	5
		Personal Relevance (PR)	Uncertainty/Nature of Science (NOS)	Critical Voice (CV)	Shared Control (SC)	Student Negotiation (SN)
1.00	Mean	20.09	13.22	18.69	15.69	20.31
	N	32	32	32	32	32
	Std.Deviation	2.798	3.957	3.605	3.856	2.681
2.00	Mean	17.00	7.67	16.67	13.67	17.00
	N	3	3	3	3	3
	Std.Deviation	2.646	3.786	4.509	3.055	3.606
Total	Mean	19.83	12.74	18.51	15.51	20.03
	N	35	35	35	35	35
	Std.Deviation	2.885	4.196	3.657	3.799	2.864

## Appendix A

**DIRECTIONS**

**1. Purpose of the Questionnaire**

This questionnaire asks you to describe important aspects of the science classroom which you are in right now. There are no right or wrong answers. Your opinion is what is wanted. Your answers will enable us to improve future science teaching.

**2. How to Answer Each Question**

On the next few pages you will find 25 sentences. For each sentence, circle only one number corresponding to your answer. For example:

		**Almost Always**	**Often**	**Some-times**	**Seldom**	**Almost Never**
In this class						
8	I ask the students questions.	5	4	3	2	1

- If you think that you *almost always* ask the students questions, circle the 5.
- If you think that you *almost never* ask the students questions, circle the 1.
- Or you can choose the number 2, 3 or 4 if one of these seems like a more accurate answer.

**3. How to Change Your Answer**

If you want to change your answer, cross it out and circle a new number, For example:

8	I ask the students questions.			3	2	1
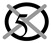
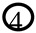

**Learning about the world (personal relevance)**		Almost Always	Often	Some-times	Seldom	Almost Never
In this class						
1	Students learn about the world outside of school.	5	4	3	2	1
2	Students’ new learning starts with problems about the world outside of school.	5	4	3	2	1
3	Students learn how science can be part of their out-of-school life.	5	4	3	2	1
In this class						
4	Students get a better understanding of the world outside of school.	5	4	3	2	1
5	Students learn interesting things about the world outside of school.	5	4	3	2	1
**Learning about science (uncertainty / nature of science)**		Almost Always	Often	Some-times	Seldom	Almost Never
In this class						
6	Students learn that science has changed over time.	5	4	3	2	1
7	Students learn that science is influenced by people’s values and opinions.	5	4	3	2	1
In this class						
8	Students learn about the different sciences used by people in other cultures.	5	4	3	2	1
9	Students learn that modern science is different from the science of long ago.	5	4	3	2	1
10	Students learn that science is about inventing theories.	5	4	3	2	1
**Learning to speak out (critical voice)**		Almost Always	Often	Some-times	Seldom	Almost Never
In this class						
11	It’s OK for students to ask me “why do I have to learn this?”	5	4	3	2	1
12	It’s OK for students to question the way I’m teaching.	5	4	3	2	1
13	It’s OK for students to complain about activities that are confusing.	5	4	3	2	1
In this class						
14	It’s OK for students to complain about anything that prevents them from learning.	5	4	3	2	1
15	It’s OK for students to express their opinions.	5	4	3	2	1
**Learning to learn (shared control)**		Almost Always	Often	Some-times	Seldom	Almost Never
In this class						
16	Students help me to plan what they’re going to learn.	5	4	3	2	1
17	Students help me to decide how well they are learning.	5	4	3	2	1
18	Students help me to decide which activities are best for them.	5	4	3	2	1
In this class						
19	Students help me to decide how much time they spend on activities.	5	4	3	2	1
20	Students help me to decide which activities they do.	5	4	3	2	1
**Learning to communicate (student negotiation)**		Almost Always	Often	Some-times	Seldom	Almost Never
In this class	
21	Students get the chance to talk to other students.	5	4	3	2	1
22	Students talk with other students about how to solve problems.	5	4	3	2	1
23	Students explain their ideas to other students.	5	4	3	2	1
In this class						
24	Students ask other students to explain their ideas.	5	4	3	2	1
25	Students listen carefully to each other’s ideas.	5	4	3	2	1
		Almost Always	Often	Some-times	Seldom	Almost Never

**The Ethics Position Questionnaire**

Please indicate if you agree or disagree with the following items. Each represents a commonly held opinion and there are no right or wrong answers. We are interested in your reaction to such matters of opinion. Rate your reaction to each statement by writing a number to the left of each statement where:

1 = Disagree strongly

2 = Disagree slightly

3 = Neither agree nor disagree

4 = Agree slightly

5 = Agree strongly

**Please provide a response to each statement.**

1. People should make certain that their actions never intentionally harm another even to a small degree.
2. Risks to another should never be tolerated, irrespective of how small the risks might be.
3. The existence of potential harm to others is always wrong, irrespective of the benefits to be gained.
4. One should never psychologically or physically harm another person.
5. One should not perform an action which might in any way threaten the dignity and welfare of another individual.
6. If an action could harm an innocent other, then it should not be done.
7. Deciding whether or not to perform an act by balancing the positive consequences of the act against the negative consequences of the act is immoral.
8. The dignity and welfare of the people should be the most important concern in any society.
9. It is never necessary to sacrifice the welfare of others.
10. Moral behaviours are actions that closely match ideals of the most “perfect” action.

1 = Disagree strongly

2 = Disagree slightly

3 = Neither agree nor disagree

4 = Agree slightly

5 = Agree strongly

**Please provide a response to each statement.**

11. There are no ethical principles that are so important that they should be a part of any code of ethics.
12. What is ethical varies from one situation and society to another.
13. Moral standards should be seen as being individualistic; what one person considers to be moral may be judged to be immoral by another person.
14. Different types of morality cannot be compared as to “rightness.”
15. Questions of what is ethical for everyone can never be resolved since what is moral or immoral is up to the individual.
16. Moral standards are simply personal rules that indicate how a person should behave, and are not to be applied in making judgments of others.
17. Ethical considerations in interpersonal relations are so complex that individuals should be allowed to formulate their own individual codes.
18. Rigidly codifying an ethical position that prevents certain types of actions could stand in the way of better human relations and adjustment.
19. No rule concerning lying can be formulated; whether a lie is permissible or not permissible totally depends upon the situation.
20. Whether a lie is judged to be moral or immoral depends upon the circumstances surrounding the action.

**Thank you for filling in both questionnaires. It is much appreciated.**
